# Investigation of a 4H-SiC Trench MOSFET with Back-Side Super Junction

**DOI:** 10.3390/mi13101770

**Published:** 2022-10-18

**Authors:** Lili Zhang, Yuxuan Liu, Junpeng Fang, Yanjuan Liu

**Affiliations:** 1 College of Electronic and Information Engineering, Shenyang Aerospace University, Shenyang 110136, China; 2 School of Integrated Circuits, Tsinghua University, Beijing 100084, China

**Keywords:** UMOS, breakdown voltage, FOM

## Abstract

In this paper, a 4H-SiC trench gate MOSFET, featuring a super junction layer located on the drain-region side, is presented to enhance the breakdown voltage and the figures of merit (FOM). The proposed structure is investigated and compared with the conventional structure with a 2D numerical simulator—ATLAS. The investigation results have demonstrated that the breakdown voltage in the proposed structure is enhanced by 21.2%, and the FOM is improved by 39.6%. In addition, the proposed structure has an increased short-circuit capability.

## 1. Introduction

Since the silicon carbide trench-gate metal oxide semiconductor field effect transistor (UMOSFET) was first reported in 1994 [1], researchers have paid significant attention to it due to the excellent material properties of SiC, including a higher critical electric field, a wider band gap and a higher electron saturation drift velocity, compared to silicon [2,3]. However, this reported structure has a serious problem because it cannot reflect the advantage of the silicon carbide material in the withstand voltage, owing to a high electric field existing in the corner of the trench gate oxide [4,5]. In order to overcome this problem, B. J. Baliga proposed a novel UMOSFET incorporated with a P+ shielding region at the bottom of the trench gate [6], which is used to protect the gate oxide from a high electric field. However, at the same time, this also increases the specific on-resistance and power loss of the device by introducing a parasitic JFET region, composed of the p-body, n-drift and P+ shielding region.

Since then, more research has been done on how to reduce the specific on-resistance and increase the breakdown voltage of the UMOSFET devices. The research findings show that there exists a tradeoff between the specific on-resistance and breakdown voltage. Particularly, the figures of merit (FOM) is utilized to indicate the compromise between the two performances, which is defined as FOM = BV^2^/R_on,sp_. Moreover, based on the existing literature, there are two domination ways used to improve the device’s performances. One is to adopt a new structure [7,8,9,10,11,12,13,14,15,16,17,18], with a mechanism that reduces the JFET resistance. The other is adopting a new fabrication process for the gate oxide [19,20,21,22,23,24,25], which increases the channel’s electron mobility to achieve a reduction in the resistance. In this paper, a new trench gate MOSFET is investigated to improve the electrical characteristics of the device.

A 4H-SiC trench gate MOSFET with a back-side super junction layer (called as BSJ-UMOS) is investigated. Compared with a conventional trench gate, MOSFET (C-UMOS), the BSJ-UMOS has a p-type region on the drain-region side, forming a floating super junction, which modulates the electric field in the N-drift region and introduces an exact peak electric field at the p-pillar/n+ substrate region junction. A comparative study between BSJ-UMOS and C-UMOS is conducted in this paper based on a 2D numerical simulator—ATLAS [26]. This paper is organized as follows. The parameters related to the device structure and simulation condition are presented in Section 2. Section 3 presents an analysis and discussion on the simulation results. Finally, we draw a conclusion in Section 4.

Note that due to the limitation of our present experimental conditions, we are unable to provide experimental results, and the simulation tools are generally used for the optimization and development of various semiconductor device structures, in order to reduce the cost of the device manufacturability and development period and, hence, lower the risk of technology transfer in an industrial environment. Moreover, simulation tools are very useful to explore the novel device architectural concept for systems with different materials. So, the aim of the simulation work is to compare the electrical characteristics of two different structures on the same terms, while not revealing the physical devices’ features.

## 2. Device Structure and Simulation Setup

**Figure 1:**
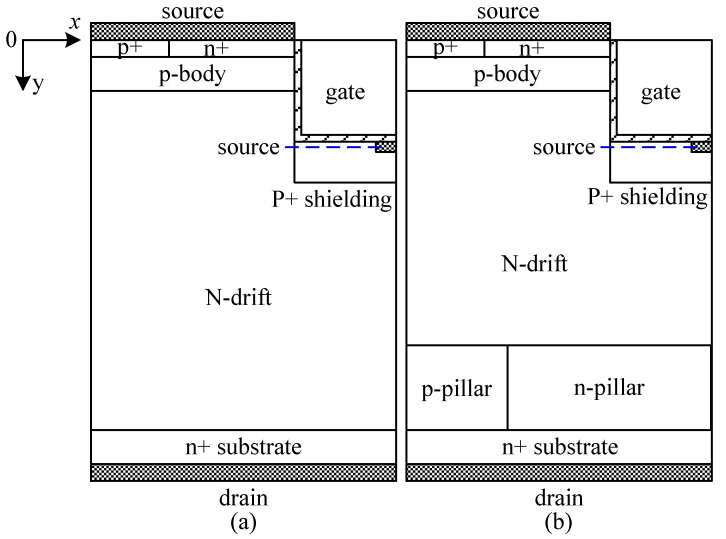
Cross-sectional schematic of a half-cell in (**a**) C-UMOS and (**b**) BSJ-UMOS.

The 4H-SiC UMOS with a back-side super junction layer (BSJ-UMOS) is as shown in Figure 1b. The drift region of BSJ-UMOS consists of two parts, which are an N-drift region and a super junction layer, composed of the p-pillar and n-pillar. The super junction layer forms extra PN junctions on the drain-region side. As a result, a new electric field peak comes into being, which can modulate the original electric field distribution and improve the breakdown voltage.

Figure 1a shows a cross-sectional schematic of the conventional UMOS (C-UMOS). Note that all the simulation parameters of the two structures are identical, except that there is an extra PN junction at the back side of the proposed structure. The gate oxide thickness is 50 nm. The other simulation parameters are presented in Table 1.

**Figure 2:**
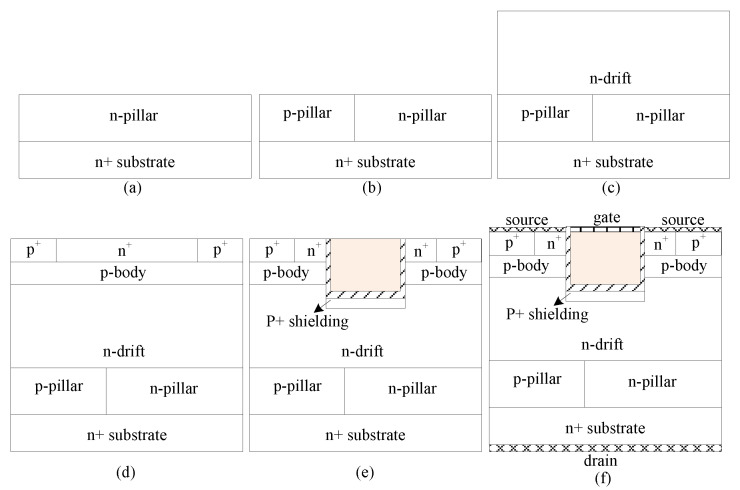
A possible fabrication process for BSJ-UMOS (**a**) growth of the n-pillar layer on n+ substrate (**b**) growth of the p-pillar layer by ion implantation (**c**) growth of the n-drift layer (**d**) growth of the p-body, n+ source and p+ contact regions (**e**) forming the trench gate structure (**f**). metalizing all contacts.

A possible fabrication process for BSJ-UMOS is shown in Figure 2. The key fabrication steps are as follows: (a) forming the n-pillar layer on the n+ wafer by epitaxial growth technology; (b) forming the p-pillar layer by ion implantation; (c) growing the n-drift layer by epitaxial growth technology; (d) forming the p-body layer by epitaxial growth technology and growing the P+ contact region and n+ source region by ion implantation; (e) forming the trench gate structure with the mature silicon carbide fabrication process; (f) metalizing all contacts and forming the gate, source, and drain electrodes. Compared with C-UMOS, such fabrication of BSJ-UMOS would be more complex, as it requires two extra process steps—growing the epitaxial n-pillar layer on the n+ substrate and forming the p-pillar layer via ion implantation.

In this paper, the electrical characteristics of the proposed device are investigated in detail, with a 2D numerical simulator—ATLAS. Since the simulated n-channel IGBT is calibrated with an experimental IGBT [26] device in [27], and these physical models have earlier been applied for 4H-SiC devices [28,29], the simulation is conducted with the same physical models and parameters as [27]. During the simulation, the following models are considered: a bandgap narrowing model (BGN), AUGER and Shockley-Read-Hall (SRH) for recombination and carrier lifetime models and doping- and temperature-dependent field mobility models (ANALYTIC) [30]. Moreover, all simulations are carried out using Fermi Dirac statistics, and Selberherr’s impact ionization model is also utilized [30]. During the simulation, the carrier lifetime in the drift region is set to 1μs, and the channel inversion mobility is 30 cm^2^/V·s.

## 3. Analysis and Discussion of Simulation Results

In this section, a comparative investigation between BSJ-UMOS and C-UMOS is carried out for the on-state, off-state and short-circuit performances. The effect of the p-pillar’s parameters on the electrical characteristics is presented in depth.

### 3.1. On and Off Characteristics

**Figure 3:**
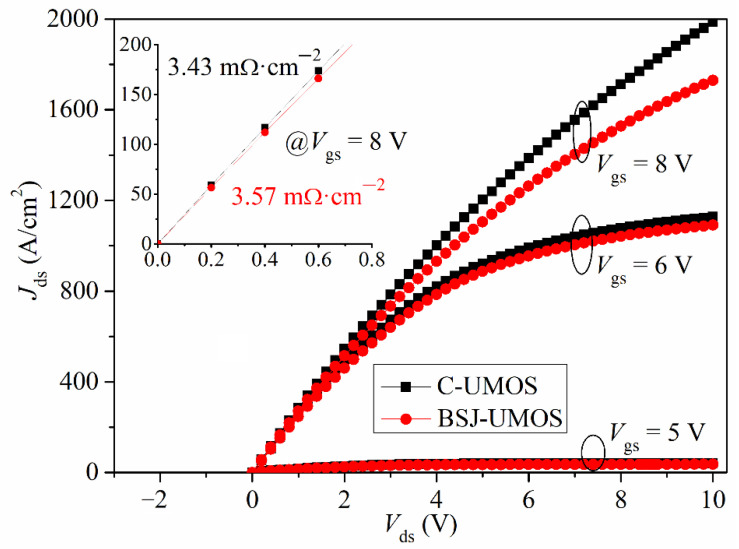
Comparison of I-V characteristic curves.

**Figure 4:**
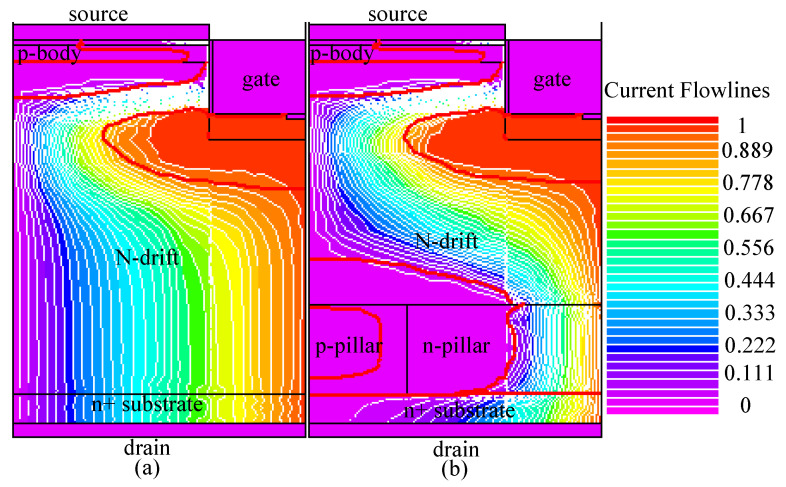
Distributions of current flowlines at *J*_ds_ = 100 A/cm^2^ in (**a**) C-UMOS and (**b**) BSJ-UMOS.

The comparison of the *I-V* characteristic between BSJ-UMOS and C-UMOS is shown in Figure 3. The *I-V* curves at *V*_gs_ = 8 V at the low drain voltage are also given in the inset of Figure 3. From this graph, it can be seen that the forward conduction performance of BSJ-UMOS is slightly degraded. At *J*_ds_ = 100 A/cm^2^ and *V*_gs_ = 8 V, the specific on-resistance *R*_on,sp_ is 3.57 mΩ·cm^2^ for BSJ-UMOS and increases about 4.08% compared with that for C-UMOS (3.43 mΩ·cm^2^). The main factor responsible for this is that the existence of the p-pillar region forms a depletion layer in the n-pillar region and narrows down the current flow path. The distribution of the current flowlines in the two structures is shown in Figure 4. The bold red lines represent the depletion layer border, indicating that the current flow path close to the drain region is reduced in BSJ-UMOS.

**Figure 5:**
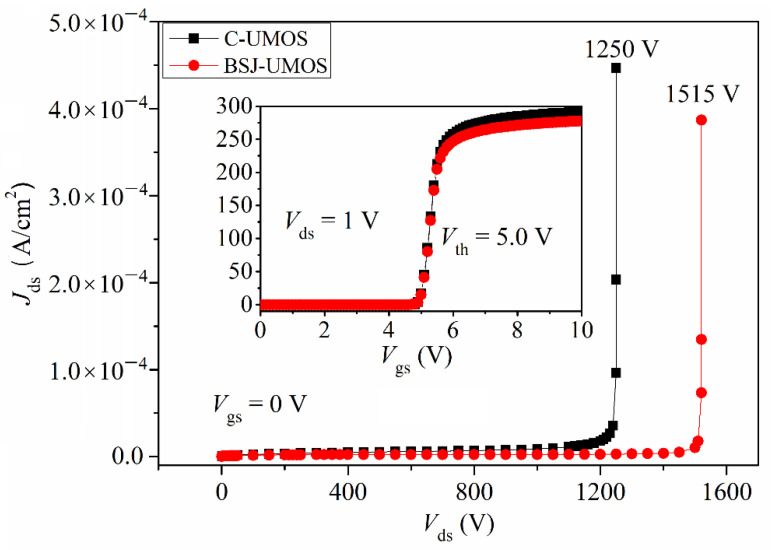
Comparisons of transfer and breakdown characteristics.

The transfer characteristic at *V*_ds_ = 1.0 V for BSJ-UMOS and C-UMOS is presented in the inset of Figure 5. Obviously, the two structures have the same threshold voltage, about 5.0 V. Moreover, it can be seen that BSJ-UMOS has a weak current drive capability because of the introduction of the p-pillar region, which narrows down the current flow path.

**Figure 6:**
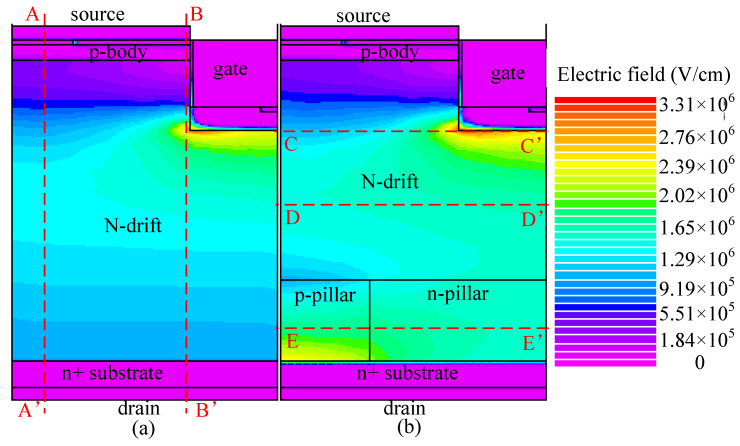
Electric field distributions at *V*_gs_ = 0 V and *V*_ds_ = BV in (**a**) C-UMOS and (**b**) BSJ-UMOS.

The breakdown characteristic is shown in Figure 5. The breakdown voltage is 1515 and 1250 V for BSJ-UMOS and C-UMOS, respectively. The breakdown voltage of BSJ-UMOS is enhanced by 21.2%. This is due to the introduction of the p-pillar/n-pillar junction, which modulates the electric field in the drift region. Figure 6 describes the electric field distribution in BSJ-UMOS and C-UMOS at *V*_gs_ = 0 V and *V*_ds_ = BV. The back-side super junction, composed of the p-pillar and n-pillar region, has two effects on the electric field. One is that the electric field in the drift region of BSJ-UMOS is more well-distributed than in C-UMOS. The other is that there is an additional peak electric field at the p-pillar/n+ substrate junction. These two factors result in the enhancement of the breakdown voltage.

**Figure 7:**
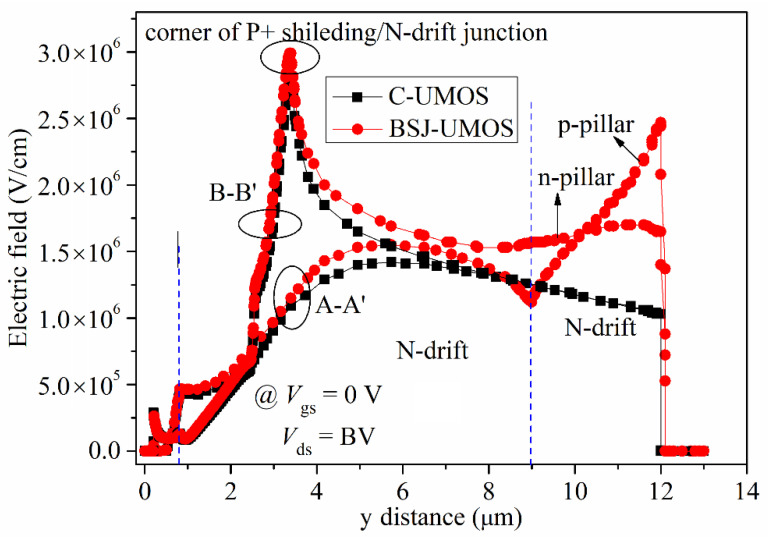
Electric field distributions of the BSJ-UMOS and C-UMOS along the AA’ (*x* = 0.5 μm) and BB’ (*x* = 2.0 μm) lines, as shown in Figure 5a.

Figure 7 shows a comparison of the electric field distribution of BSJ-UMOS and C-UMOS in the vertical cross-section. From this graph, it can be seen that C-UMOS has only a peak electric field at the P+ shielding/N-drift region junction, and the electric field reduces from the source to the drain side. By contrast, BSJ-UMOS has a two peak electric field. One is almost the same as that of C-UMOS, and the other is at the p-pillar/n+ substrate junction. Moreover, due to the introduction of the p-pillar/n-pillar junction, the electric field intensity from the P+ shielding to the n+ substrate region is well-distributed in BSJ-UMOS. In addition, these lead to an improved breakdown characteristic.

**Figure 8:**
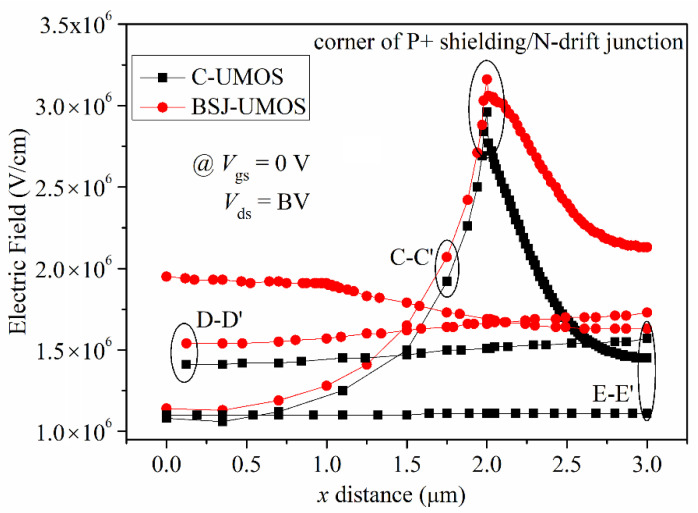
Electric field distributions of BSJ-UMOS and C-UMOS along CC’ (*y* = 3.4 μm), DD’ (*y* = 6.0 μm) and EE’ (*y* = 11 μm) lines, as shown in Figure 5b.

The electric field distribution of BSJ-UMOS and C-UMOS in the lateral cross-section is shown in Figure 8. Due to the existence of the back-side super junction layer, the electric field in the lateral cross-section is significantly strengthened, especially in the p-pillar and n-pillar region, in which the electric field intensity is about double, effectively improving the breakdown voltage of the device.

Next, the performance of BSJ-UMOS and C-UMOS in terms of the FOM values is compared, which indicates the tradeoff between the off-state characteristic (BV) and on-state characteristic (*R*_on,sp_). The FOM values are 652.24 and 467.12 MW/cm^2^ for BSJ-UMOS and C-UMOS, respectively. In addition, the FOM of BSJ-UMOS is improved by 39.6%, which indicates the significant influence of the back-side super junction layer on the devices’ performances.

### 3.2. Other Performances

In this section, the other performances, gate charge and capacitance, are investigated.

**Figure 9:**
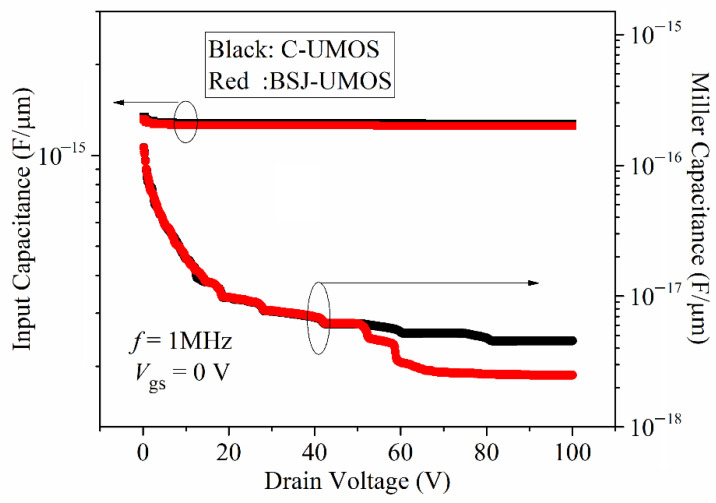
Comparisons of the parasitic capacitance in C-UMOS and BSJ-UMOS.

**Figure 10:**
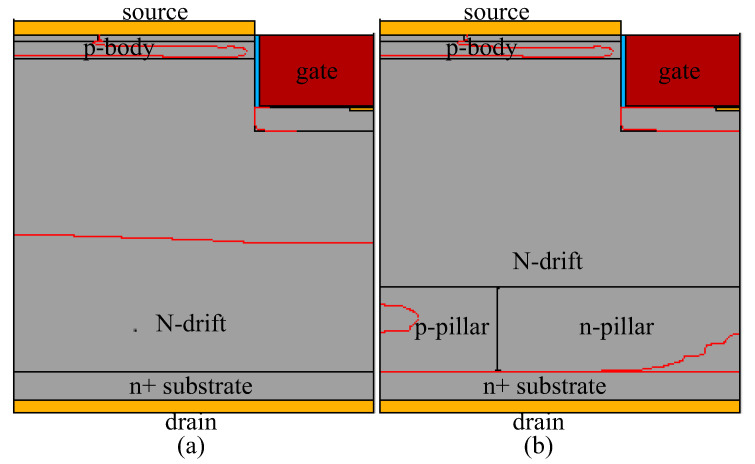
Depletion distributions of (**a**) C-UMOS and (**b**) BSJ-UMOS at *V*_ds_ = 70 V and *V*_gs_ = 0 V.

Figure 9 plots the parasitic capacitance performance (input capacitance *C*_ies_ and miller capacitance *C*_res_), indicating that the two structures have almost identical parasitic capacitance when the drain voltage is lower than 60 V. However, when the drain voltage is larger, the BSJ-UMOS has lower miller capacitance, mainly due to the p-pillar making the depletion wider in the n-drift region, as shown in Figure 10. In addition, this is due to the p-pillar being accelerated the depletion of the n-drift region.

**Figure 11:**
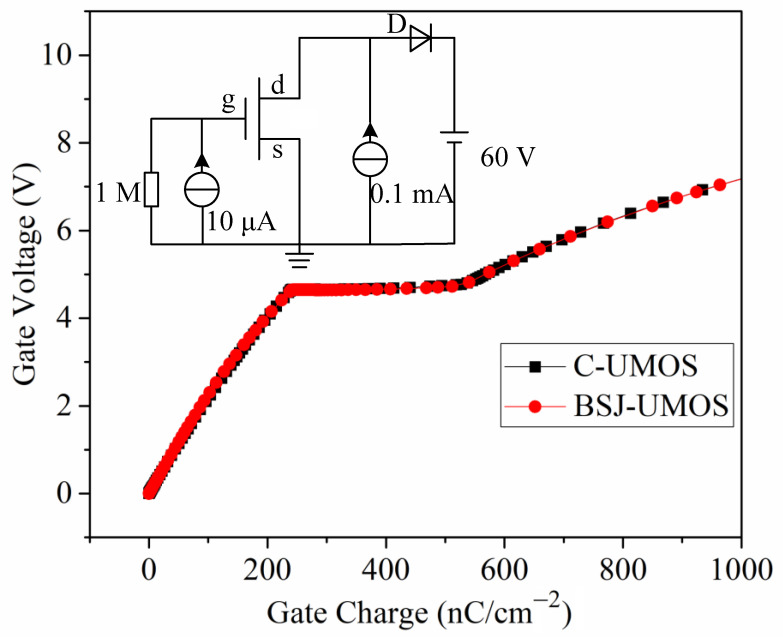
Comparisons of the gate charge performances.

The gate charge characteristics and its test circuit are shown in Figure 11. From this, it can be seen that the two structures have an identical gate charge, because the gate charge is mainly dependent on the front structure, and the two structures have the same front structure and parameters.

### 3.3. Parameter Influence

In this section, we investigate the effect of the concentration of the p-pillar and n-pillar on the electrical characteristics, mainly including the breakdown voltage, specific on-resistance and FOM.

**Figure 12:**
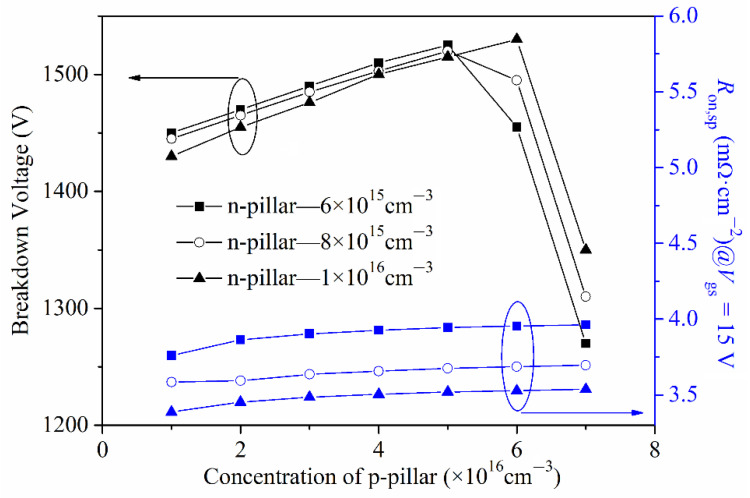
Effect of concentration of the p-pillar and n-pillar on the BV and *R*_on,sp_.

**Figure 13:**
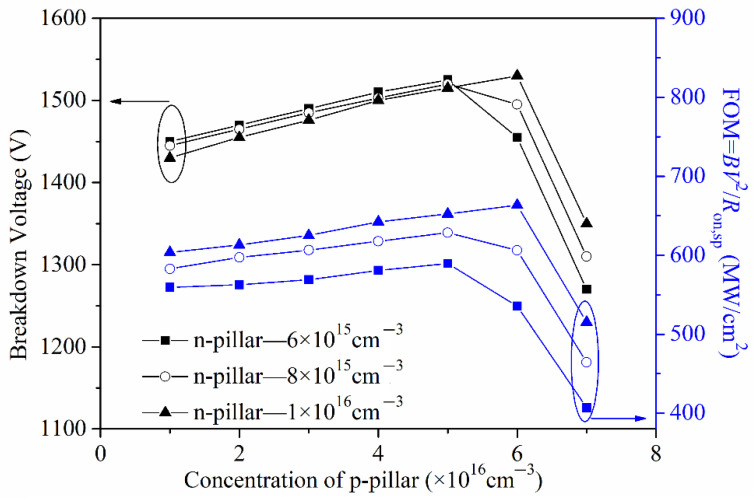
Effect of the concentration of the p-pillar and n-pillar on the BV and FOM.

Figure 12 shows the effect of the concentration of the p-pillar and n-pillar on the BV and *R*_on,sp_. As we can see from this, with the increasing of the n-pillar’s concentration, the specific on-resistance *R*_on,sp_ decreases, and the breakdown voltage is almost unchanged when the p-pillar’s concentration is lower 6 × 10^16^ cm^−3^ and then increases. The increasing of the n-pillar’s concentration makes the depletion layer narrower and the current flow path wider, leading to a reduction in *R*_on,sp_. With the increasing of the p-pillar’s concentration, the BV first increases to the maximum value and then decreases, while the *R*_on,sp_ is almost unchanged. This is due to the charge in the back-side super junction layer being gradually balanced and then imbalanced, with the increasing of the p-pillar’s concentration. As we can see from Figure 13, the FOM has the same changing trend as the BV, and the changing reason is also the same.

### 3.4. Charge Imbalance and Design Windows

**Figure 14:**
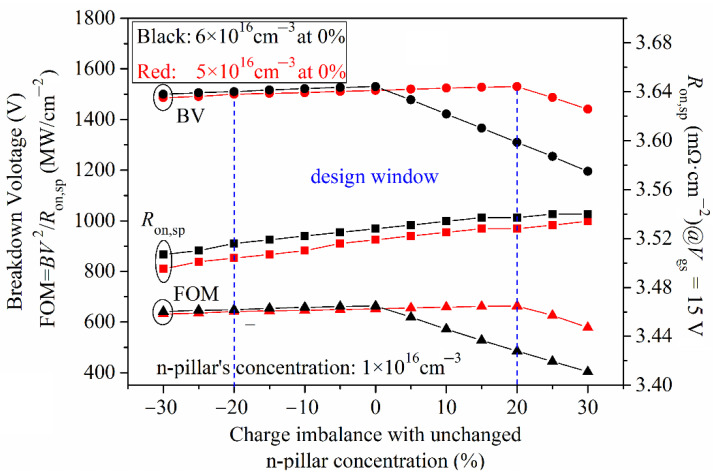
Relationship curves of BV, *R*_on,sp_ and FOM versus charge imbalance for BSJ-UMOS.

For a super junction structure, the charge imbalance is unavoidable and is a serious problem that has to be mentioned, especially for silicon carbide device processing technology. Figure 14 shows the relationship curve of the BV, *R*_on,sp_ and FOM versus the charge imbalance in BSJ-UMOS. Ideally, the highest BV can be obtained when the charge is balanced. However, when the charge balance is broken, the breakdown voltage begins to decline from the highest value. A charge imbalance, changing from negative to positive, means that the p-pillar’s concentration increases, the depletion layer narrows down and, thus, the *R*_on,sp_ increases, as Figure 14 shows. From Figure 13, we can see that the highest BV and FOM are achieved when the concentrations of the p-pillar and n-pillar are 6 × 10^16^ cm^−3^ and 1 × 10^16^ cm^−3^, respectively. However, from Figure 14, it can be seen that the design window is wider when the concentration of the p-pillar is 5 × 10^16^ cm^−3^. In this design window, when the charge imbalance changes from –20% to 20%, the breakdown voltage is about 1500 V, and the FOM ranges from 642.12 to 663.52 MW/cm^2^, which is easy for the control of the process technology.

### 3.5. Short-Circuit Capability

**Figure 15:**
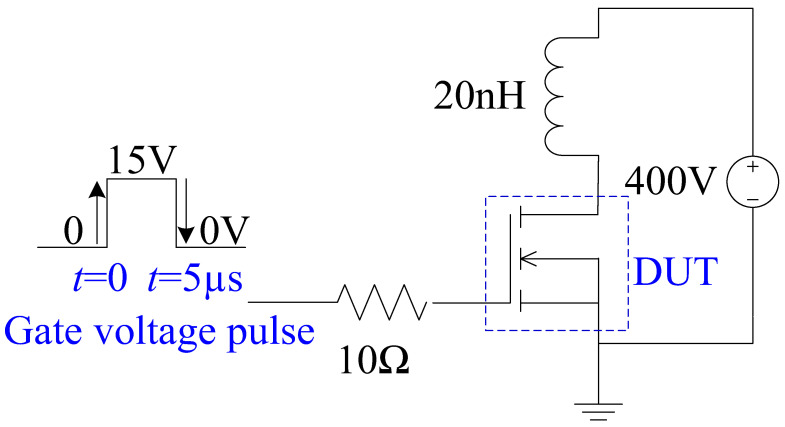
Test circuit of the short-circuit performance.

**Figure 16:**
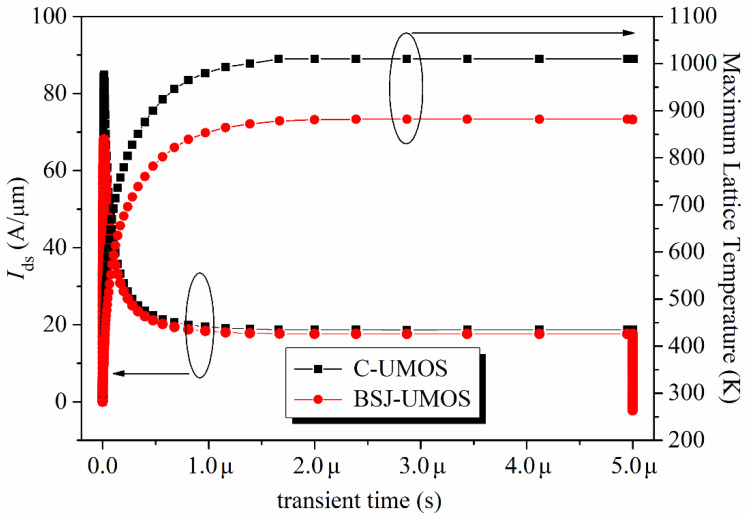
Maximum lattice temperature and drain current for the short-circuit case.

The short-circuit case is defined as follows: when the 4H-SiC MOSFET operates in the conduction mode, the high-voltage source is directly biased at the drain electrode, due to the shorting of the load [31,32]. At the same time, a high drain current flows through the device with a high drain voltage, which generates a high power loss and makes the device’s temperature increase. From the I-V performance (shown in Figure 3), the BSJ-UMOS has a lower saturation current due to the introduction of the p-pillar, meaning an increased short-circuit capability. The test circuit of the short-circuit characteristic is shown in Figure 15, and the simulation results are presented in Figure 16, in which the drain current pulse is caused by the inductance load. As expected, compared with C-UMOS, BSJ-UMOS has a lower drain current and minimum lattice temperature during the short-circuit case, since the existence of the p-pillar shrinks the current flow path. Owing to the lower lattice temperature, BSJ-UMOS has an improved short-circuit capability.

## 4. Conclusions

A 4H-SiC trench gate MOSFET structure with a super junction layer (composed of the p-pillar and n-pillar) on the drain-region side is investigated in detail and compared with a conventional trench gate, MOSFET. The investigation results demonstrate that the proposed structure can significantly enhance the breakdown voltage, with a slight degradation of the specific on-resistance. The introduction of a back-side super junction layer can modulate the electric field in the drift region and introduce an exact peak electric field at the p-pillar/n+ substrate junction, resulting in a significant improvement in the FOM. Moreover, BSJ-UMOS has an increased short-circuit capability due to a lower saturation current.

## Figures and Tables

**Table 1:** Structural parameters of the simulated devices.

Parameters	C-UMOS	BSJ-UMOS
Concentration of n+/p+ region (cm^−3^)	1.0 × 10 ^ 19 ^	1.0 × 10 ^ 19 ^
Concentration of p-body region (cm^−3^)	1.0 × 10 ^ 17 ^	1.0 × 10 ^ 17 ^
Thickness of p-body region (μm)	0.6	0.6
Width of trench gate (μm)	1.0	1.0
Depth of trench gate (μm)	2.5	2.5
Concentration of P+ shielding region (cm^−3^)	1.0 × 10 ^ 18 ^	1.0 × 10 ^ 18 ^
Concentration of N-drift region (cm^−3^)	4.0 × 10 ^ 15 ^	4.0 × 10 ^ 15 ^
Thickness of N-drift region (μm)	11.2	8.2
Concentration of p-pillar (cm^−3^)	-	5 × 10 ^ 16 ^
Thickness of p-pillar region (μm)	-	3
Width of p-pillar region (μm)	-	1
Concentration of n-pillar region (cm^−3^)	-	1 × 10 ^ 16 ^
Thickness of n-pillar region (μm)	-	3
Width of n-pillar region (μm)	-	2
Concentration of n+ substrate region (cm^−3^)	1 × 10 ^ 19 ^	1 × 10 ^ 19 ^
Width of a half cell (μm)	3	3

## Data Availability

Not applicable.
